# Heterothermy as a mechanism to offset energetic costs of environmental and homeostatic perturbations

**DOI:** 10.1038/s41598-021-96828-0

**Published:** 2021-09-24

**Authors:** Javier Omar Morales, Nikki Walker, Robin W. Warne, Justin G. Boyles

**Affiliations:** 1 School of Biological Sciences, Southern Illinois University, Carbondale, IL 62901 USA; 2 Cooperative Wildlife Research Laboratory, Southern Illinois University, Carbondale, Illinois 62901 USA

**Keywords:** Behavioural ecology, Homeostasis

## Abstract

Environmental and biotic pressures impose homeostatic costs on all organisms. The energetic costs of maintaining high body temperatures (*T*_b_) render endotherms sensitive to pressures that increase foraging costs. In response, some mammals become more heterothermic to conserve energy. We measured *T*_b_ in banner-tailed kangaroo rats (*Dipodomys spectabilis*) to test and disentangle the effects of air temperature and moonlight (a proxy for predation risk) on thermoregulatory homeostasis. We further perturbed homeostasis in some animals with chronic corticosterone (CORT) via silastic implants. Heterothermy increased across summer, consistent with the predicted effect of lunar illumination (and predation), and in the direction opposite to the predicted effect of environmental temperatures. The effect of lunar illumination was also evident within nights as animals maintained low *T*_b_ when the moon was above the horizon. The pattern was accentuated in CORT-treated animals, suggesting they adopted an even further heightened risk-avoidance strategy that might impose reduced foraging and energy intake. Still, CORT-treatment did not affect body condition over the entire study, indicating kangaroo rats offset decreases in energy intake through energy savings associated with heterothermy. Environmental conditions receive the most attention in studies of thermoregulatory homeostasis, but we demonstrated here that biotic factors can be more important and should be considered in future studies.

## Introduction

Animals balance interrelated energy, water, and thermoregulatory demands using coupled physiological and behavioral mechanisms. While these homeostatic responses are essential to function and survival, they can incur energetic, resource, or fitness costs^1^ especially when environmental perturbations or biotic interactions push the animal well beyond a homeostatic range^2^. In endotherms, high energetic and water costs mediate physiological and behavioral thermoregulation in ways that directly affect how an animal interacts with its environment. Conversely, extrinsic environmental factors like predation that change foraging or exploratory behaviors might impose challenges to homeostatic control if energy or water intake decreases^3^.

Nominally homeothermic mammals often lessen homeostatic control of thermoregulation when faced with energy limitations. In fact, heterothermy to varying degrees is increasingly viewed as the norm rather than an exception among endotherms^4–8^. In small endotherms with high surface area to volume ratios, even small deviances from constant body temperature (*T*_b_) can lead to considerable energy savings^9^. Heterothermy reduces energetic needs and possibly exposure to predators^10^ but is not a completely cost-free strategy. Endotherms might be highly sensitive to deviations in *T*_b_, at least at the tissue level^11^, and temperature directly affects the processes and chemical reaction rates involved with metabolism^12,13^. Further, more time spent at heterothermic *T*_b_s might mean less time spent undertaking important tasks like foraging, mate searching, or defense of a territory. Taken together, an individual animal must trade-off fitness benefits of remaining sheltered from predators or adverse environmental conditions against fitness costs of allowing *T*_b_ to vary. Measurements of variation in thermoregulation might, therefore, serve as a tangible proxy for estimating the strength and importance of environmental conditions on homeostatic regulation.

Two of the main determinants of heterothermy in endotherms are environmental conditions and activity^14^. For most endotherms, variation in *T*_b_ is usually lowest within the thermoneutral zone, increasing as environmental temperatures either decrease below the lower critical limits of thermoneutrality or increase above the upper critical limits of thermoneutrality. In large part, this response is associated with physiological control of metabolic heat production. Conversely, foraging, locomotion, evasion of predators, and any number of other behaviors require an increase in metabolic rates to power these activities, indirectly causing an increase in metabolic heat production. This usually leads to a decrease in heterothermy except when activity occurs in extreme heat. Under some conditions, metabolic heat produced as a byproduct of activity might even substitute for metabolic heat produced to maintain a high and constant *T*_b_^15^. Unfortunately, it can be difficult to disentangle the physiological and behavioral aspects of endothermic thermoregulation, especially under natural conditions.

**Figure 1:**
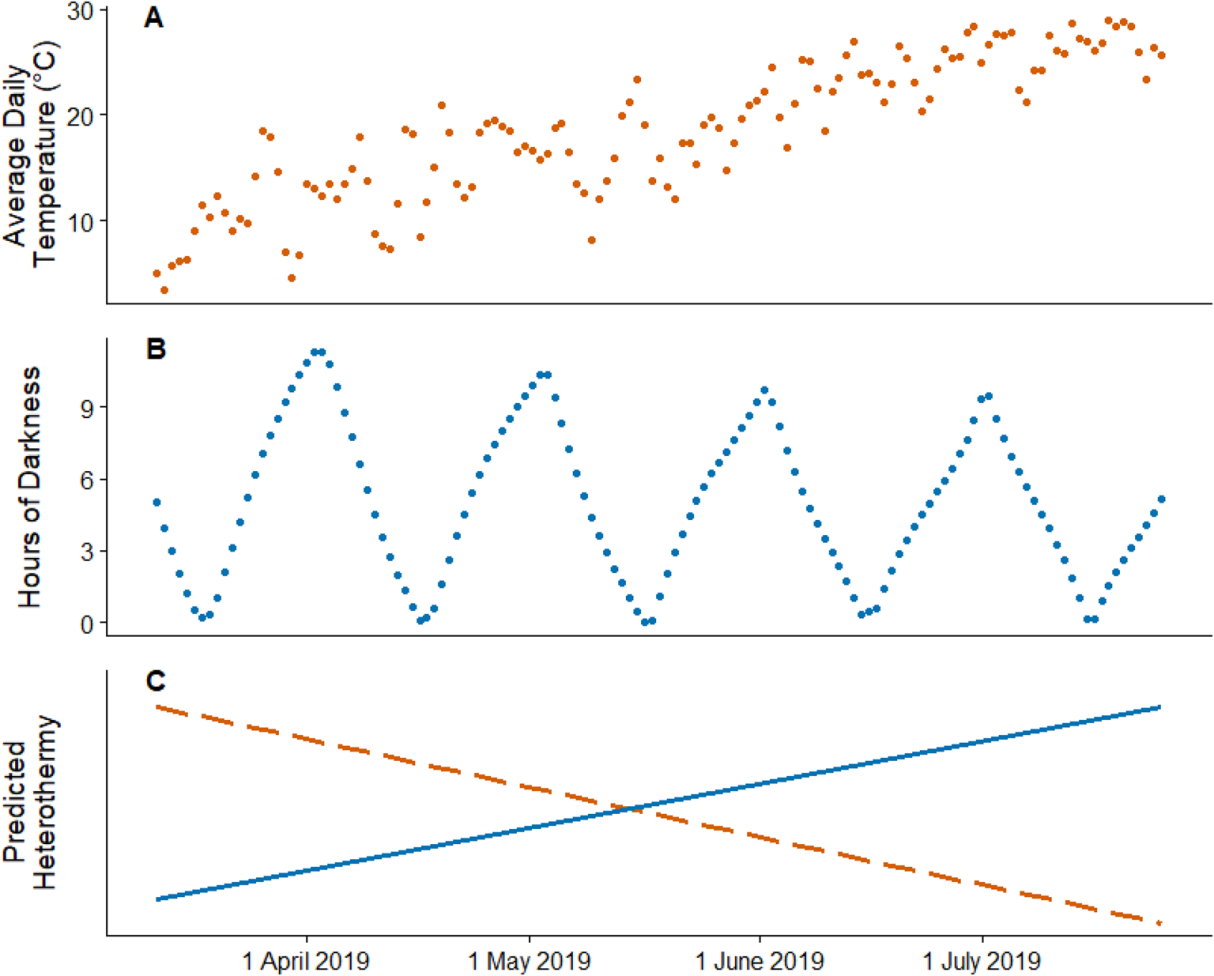
Both environmental temperatures and activity affect thermoregulation in endotherms, though in potentially contrasting ways. Activity in nocturnal desert rodents is known to be strongly correlated to the lunar cycle, as rodents forage most intensely during periods when the moon is below the horizon and visual predators are less effective. The opposing trends in temperature and hours of darkness across the study period allow us to separate their respective effects on thermoregulation in banner-tailed kangaroo rats (*Dipodomys spectabilis*) on the Sevilleta National Wildlife Refuge. Temperatures generally increased across the study period (**A**), while the hours of darkness (defined here as the time between sunset and sunrise when the moon was below the horizon) generally decreased across the study period (**B**). Our study took place between roughly spring equinox and summer solstice, so the length of night decreased over the study period. At any point of the lunar cycle, there were more hours of true darkness at the beginning of the study period than at the end. For example, there were approximately 11.3 h of darkness on the night of the first new moon of the study period and 9.5 h on the night of the last full moon. Thus, if environmental temperature is more important in determining variation in body temperature, we predicted heterothermy should decrease across the study period (**C**; dashed line). If activity periods are more important, we predicted heterothermy should increase across the study period (**C**; solid line). We predicted the administration of corticosterone would strengthen the effect of moonlight but have little on the response to environmental temperatures.

Our goal was therefore to do exactly that: separate the effects of environmental temperatures and activity on thermoregulation in an endotherm. To do so, we measured *T*_b_ in banner-tailed kangaroo rats (Heteromyidae; *Dipodomys spectabilis*) across a 5-month study period from spring through summer. This study system provided an opportunity to test contrasting predictions about the effects of environmental temperatures and activity on thermoregulation (Fig. 1). From spring to summer, environmental temperatures increase and become less variable, which should theoretically be met with a decrease in heterothermy by a nocturnal rodent (Fig. 1A,C). Conversely, if activity is an important determinant of *T*_b_, we would expect heterothermy to increase across the course of the study period as the available nocturnal foraging time decreases with seasonally shortened nights during the summer (Fig. 1B,C). Additionally, moon phase could affect monthly activity and heterothermy patterns because increased moon illumination increases predation risk for many nocturnal tetrapods sharing habitats with visual predators^16–19^. Considering that moon phase is a regular and reliable cue of risk, adaptive behavioral responses (and potentially physiological responses, see below) are expected in such animals^18,20^. For example, many desert rodents, especially open-area foraging species, respond to moon illumination by avoiding activity outside the burrow under moonlight^21^ when nocturnal visual predators are most active^16,18,19,21,22^.

To help further differentiate between these predicted outcomes, we also exposed some individuals to homeostatic perturbation using chronic, but mild disruption to hypothalamus–pituitary–adrenal (HPA) axis function by administering corticosterone (CORT) constantly over the entire study period via silastic implants. Daily and seasonal cycling of CORT regulates metabolic and behavioral processes associated with foraging, exploratory behaviors, and predator avoidance^23–25^. Additionally, small mammals often exhibit increased circulating CORT levels around full moon, which is thought to represent a preparatory response to a highly reliable cue of elevated predation risk^20,26^. Elevated CORT levels in general, and during full moons, increase anxiety and risk avoidance behaviors in diverse animals that commonly reduce foraging and increase refuge use^20,25,27^. We therefore predicted that chronically administered CORT should cause treatment individuals to be more sensitive to cues of predation risk and therefore accentuate any heterothermic thermoregulatory responses to seasonal shifts in daylight periods and moonlight.

## Materials and methods

### Ethical statement

The study was approved by the Southern Illinois University IACUC (permit protocol #17-008) and all experiments were conducted under approval by the appropriate authorities (Sevilleta National Wildlife Refuge Permit #18_023R, and New Mexico Department of Game and Fish permit # 3706). All kangaroo rat surgeries were performed by a licensed veterinarian of the state of New Mexico and in accordance with the ARRIVE guidelines (https://arriveguidelines.org/) as well as the National Institute of Health’s (NIH) guidelines on experimental animals (https://oacu.oir.nih.gov/).

### Study site and species

We conducted this experiment between March and August 2019 at the Sevilleta Long-Term Ecological Research Site (34°20′15.44″, − 106°41′56.32″, 1400 m AMSL) within the Sevilleta National Wildlife Refuge in New Mexico, USA. The Sevilleta, found in the Chihuahuan Desert, is dominated by patches of black and blue gramma grasses, creosote scrub, and forbs^28^. The average precipitation in spring and summer months is ~ 7 cm with monsoon seasons from July through September, raising average precipitation to 13–14 cm. During the study, daily minimum temperatures ranged from − 7.5 to 20 °C. There was a total of 4.1 cm of rain between March and April and 40.2 cm between June and July in 2019. A large storm front passed through central New Mexico in mid-April, dropping minimum temperatures by almost 10 °C for several days.

The banner-tailed kangaroo rat is a nocturnal granivorous rodent native to deserts of the southwestern United States and northern Mexico^29^. Kangaroo rats generally prefer open habitats for foraging^30^ and presumably to better assess the risks of predation^21^. Like many desert rodents, they restrict foraging under bright moonlight^31,32^. Other heteromyid rodents use adaptive heterothermy to various degrees, including torpor in some species^33,34^.

### Data collection

We captured kangaroo rats on 11 and 12 March 2019 (N = 26, 19 males and 7 females, 138.4 ± 14.9 g) with Sherman traps baited with sunflower seeds. We transported all kangaroo rats to the University of New Mexico Sevilleta Field Station for surgeries conducted by a licensed veterinarian and veterinarary technician. Under clean conditions, we induced anesthesia with 5% isoflurane in a plastic container and then maintained it using 1–1.5% isoflurane delivered via a mask. The veterinarian implanted temperature-sensitive dataloggers intraperitoneally and PIT tags and silicone pellets to deliver CORT subcutaneously between the scapulae (see below for details of loggers and implants). They sutured the abdominal walls and skin separately using 5/0 monocryl and treated surgical incisions with a 1 ml NaCl solution. We sealed the incision site for the PIT tags and silicone rods using veterinary-grade cyanoacrylate (Vetbond Tissue Adhesive; 3M) and injected animals with Ketoprofen and Baytril to limit inflammation and infection (both at 5 mg/kg). Gentocin ophthalmic ointment was used to combat eye dryness. The animals recovered on a heating pad set to 40.5 °C. We held all animals overnight with *ad lib* food and water before releasing them at their burrows the following morning.

We measured *T*_b_ at 5-min intervals with temperature-sensitive dataloggers (iButtons, Model DS1925, Maxim Semiconductor, Dallas, TX) set to a resolution of 0.0625 °C (accuracy: ± 0.5). Before implantation, we coated the iButtons in a biologically inert wax. At approximately 3.1 g when coated, the dataloggers weighed less than 3% of the average weight of the experimental animals. The iButtons were calibrated at 37 °C (± 0.02 °C) in a circulating water bath.

We split the 26 kangaroo rats into a CORT treatment group (n = 13) or control group that received a blank implant (n = 13). CORT implants consisted of crystalline corticosterone (C2505, Sigma Chemical Co., St. Louis, MO) mixed into silastic adhesive (polydimethylsiloxane, Dow Corning) and shaped into rods^35–37^. We cut the silastic implants into 3 × 14 mm rods that contained 22.4 mg of CORT each, which represented a dose of approximately 160 mg/kg BW in kangaroo rats. This is a moderate dose for rats, shown to alter HPA axis function^38–40^. In vitro tests demonstrated these implants had a sustained CORT release of approximately 36 µg/cm/day for at least 42 days, following an initial high release during the first week (see supplementary materials for full details). The estimated in vitro release rate suggests these implants should be active for a year or more, and similarly constructed silastic implants used for steroidal regulation of human and mammalian reproduction are effective for sustained hormone delivery that can extend beyond 300 days^41–43^. Over these longer periods, we would expect compensatory physiological adjustments to the production of endogenous CORT as well as receptor expression along the signaling pathway^44,45^. Finally, it should be noted that our aim was to perturb HPA axis function associated with season and moonlight in nocturnal rodents through a constant release of CORT at a low to moderate dose, but not induce chronic stress response states^38–40^.

We also validated the effects of these implants in vivo by measuring fecal corticosterone metabolites (FCM) in banner-tailed kangaroo rats on a nearby study site. We initially captured these validation animals in May and June 2019 and collected feces for baseline values before implanting them with the same control (n = 13) and CORT implants (n = 12) described above. Over the course of approximately 7 weeks, we opportunistically recaptured the animals several times and again collected feces for analysis. We limited the sample to individuals for which we had at least one fecal sample before implantation and one after. The fecal pellets were stored at − 20 °C until we conducted analysis by ELISA (DetectX® Corticosterone EIA Kit, Arbor Assay, Ann Arbor, MI, USA). To conduct ELISA, we first homogenized 2–3 pellets from each sample with a mortar and pestle. We then mixed 0.05 g with 0.5 ml of 80% ethanol in an eppendorf tube, mixed the samples on a multivortexer for 30 min, and then centrifuged them for 15 min at 5000 rpm^46,47^. We diluted supernatants to 1:10 with assay buffer, which were stored at − 20 °C until ELISA analysis.

Near the end of the summer (27 and 28 July 2019) we attempted to recapture all animals in the main study to recover iButtons. We also collected blood samples to determine if chronic exposure to CORT, as supplied by the implants, caused changes in HPA axis function. We used the stress of trapping and handling to induce an acute stress response, expecting altered HPA axis function to be apparent through attenuated CORT responses to such an acute stressor. While few studies using CORT manipulations extend beyond 2–3 weeks, there is evidence that chronically elevated CORT alters HPA axis function through changes to transcriptional activity of corticotropin releasing factors and glucocorticoid receptors^44,48,49^. Therefore, a shift in HPA responses to acute stressors among the CORT-treated animals would represent lasting effects on HPA axis function shaped by nearly 5 months of chronic exposure to elevated CORT and would provide further evidence the implants had the desired effect. After we transported the animals to the lab, we collected blood samples for quantification of CORT by ELISA (DetectX® Corticosterone EIA Kit, Arbor Assay, Ann Arbor, MI, USA). Following the manufacturer’s protocol, we treated 5 μl of plasma per sample with a dissociation agent to detect total CORT at a dilution of 1:100. We assayed all samples on a single plate.

We then euthanized the animals using a portable CO_2_ chamber modified from the methods of Ellis^50^. After euthanasia, animals were necropsied to remove iButtons and body composition was measured with a quantitative magnetic resonance (QMR) imaging machine ﻿(Echo-MRI-B, EchoMedical Systems, Houston, Texas) to determine if any changes in thermoregulatory patterns led to detectable changes in body composition. QMR is a highly accurate tool for imaging animal body composition^51–53^, providing measurements of tissue types (fat, lean, and water masses) independent of an animal’s state, body mass, or allometric proportions. Unfortunately, the QMR was not available at the time of the initial captures, so we only have post-experiment measurements of body condition. Body masses of treatment and control animals were indistinguishable at the time of initial capture (control: 138.3 ± 3.8 g; treatment: 139.0 ± 3.3 g). To measure body composition, we placed animals in a plastic bag after iButton removal and then into a clear holding tube capped with a stopper to prevent shifting inside the QMR. We used an antenna probe designed for animals between 50 and 500 g and conducted three successive scans on each animal. Each replicate scan took ~ 2.5 min at a room temperature of 21.5 °C. For our analysis, we use only percent body fat because it was highly correlated to the other measures of body composition. A known oil standard was used to calibrate the readings before any animals were scanned. We collected endoparasites for an additional study, and then deposited the specimens in the University of New Mexico Museum of Southwestern Biology.

### Data analysis

We used data collected with iButtons to detail variation in *T*_b_ using the Heterothermy Index (HI)^4^, which is analogous to standard deviation, but mean temperature is replaced by the more biologically relevant modal temperature (*T*_b-mod_) to represent the active *T*_b_^54^. As such, HI can be interpreted as an integrated measure of how much time and to what degree an animal spends with *T*_b_ other than their active temperature. Larger HI values indicate larger deviations from strict homeothermy, either when *T*_b_ decreases well below *T*_b-mod_ or when *T*_b_ remains below *T*_b-mod_ for longer periods. In this case, we calculated a single active *T*_b-mod_ for all *T*_b_ measurements between 23:00 and 03:00 because banner-tailed kangaroo rats are nocturnal and the highest *T*_b_s recorded in this study occurred at night. This period was used only for calculating *T*_b-mod_. We then calculated a daily HI for each animal on each 24-h period of the 135-day study (from 12:00 to 12:00 because the active period is at night). Thus, the calculated HI values account for variation in *T*_b_ during both the active and inactive phases. We also did post-hoc analyses of the minimum *T*_b_ to further describe thermoregulatory patterns.

Body temperature was almost always lowest throughout the day when kangaroo rats were in their burrows, and almost always highest at night when kangaroo rats were more likely to be active. As a simple metric of daily activity periods, we defined the start of activity as when *T*_b_ first increased to within 0.5 °C of *T*_b-mod_ within a night, and the cessation of activity as when *T*_b_ last decreased to more than 0.5 °C below *T*_b-mod_. This period coincided well with the known nocturnal activity period for this species (approximately between 18:00 and 4:00)^31,55^, so we believe it to be a reliable indicator of activity. A sensitivity analysis supported 0.5 °C as an appropriate cut-off because the calculated activity period was insensitive to any cut-off value below 1 °C but highly sensitive to values above 1 °C. The active period of most nocturnal rodents is comprised of many short bouts of activity away from the burrow. We attempted to use the same cut-off value to define individual foraging bouts but were unsuccessful. We suspect this would work better for diurnal species, especially in the desert, that would also be exposed to intense solar radiation while foraging. Finally, we calculated the relationship between the beginning of the active period and complete darkness and the end of the active period and sunrise. We used Package suncalc (Version 0.5.0) in R (Version 3.6.1) to estimate time of complete darkness and sunrise on the study site. During a waning phase, the moon is below the horizon at sunset and rises during the night. Thus, complete darkness occurs as soon as the effect of the sun disappears. During a waxing phase, the moon is above the horizon when the sun sets, and the moon sets at some point during the night. Thus, darkness occurs after both the sun and moon have set. Around the full moon, the moon can remain above the horizon all night.

We conducted repeated measures analyses of HI values and activity periods using mixed linear models implemented in JMP Pro (Version 15, SAS Inc., Cary, NC. 1989-2020) to account for repeated daily observations of each individual. We determined the best covariance structure for each analysis using Akaike Information Criterion (AIC_c_) to account for correlation between and within animals. In analyses of HI values, we included day, treatment, and a treatment*day interaction as fixed effects and individual as a random effect. In the activity analysis, we analyzed the beginning of the active period, the end of the active period, and total active period separately. In each model, we included treatment, moon phase, and a treatment*moon phase interaction as fixed effects and individual as a random effect. We used a two-tailed t-test to assess differences between treatments in percent fat at the end of the experiment for all animals recaptured. Finally, we used a t-test to analyze plasma CORT results. Results are reported as least squares means estimates ± standard error.

## Results

**Figure 2:**
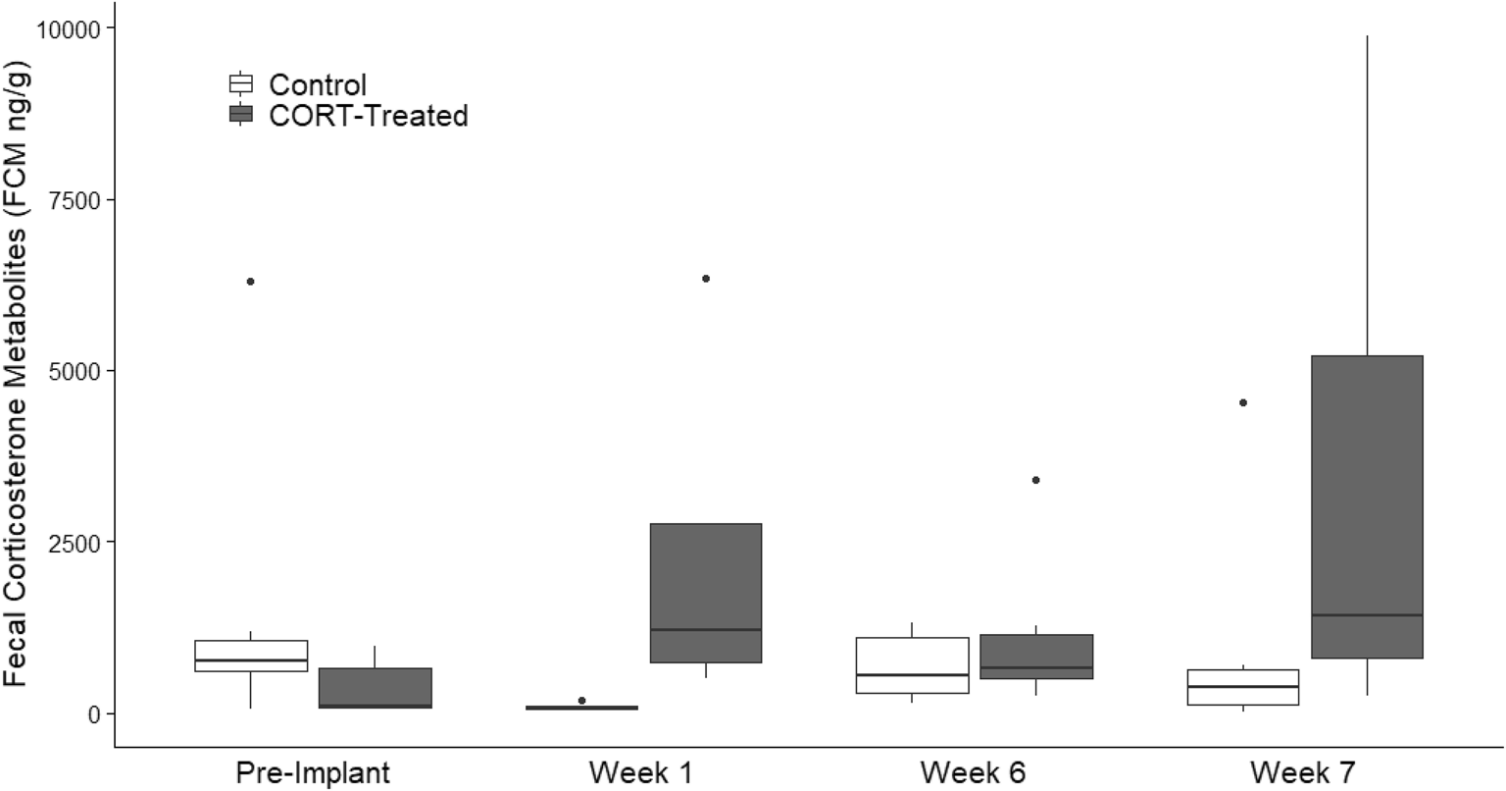
Fecal corticosterone metabolites of banner-tailed kangaroo rats that we implanted subcutaneously with either blank silastic rods (controls) or rods filled with crystalline corticosterone at a dose of 160 mg/kg BW. We captured kangaroo rats during the first and several weeks after implantation to collect fecal samples for CORT analysis.

We recaptured 15 of 26 implanted animals (11 males and 4 females) on the main study site. Of these, an iButton was unrecoverable from one animal, and in three others the iButtons shifted outside the body cavity into the thigh. Because these data did not represent core temperatures, we excluded them from analyses. In total, we were able to analyze *T*_b_ data for 11 animals (8 males and 3 females) and body condition and CORT data for 14 of 15 recaptured animals. The validation study verified CORT implants performed as expected. Fecal corticosterone metabolites were elevated up to 7 weeks after implantation in CORT-treated kangaroo rats compared to controls (F_1, 20.8_ = 7.6, *p* = 0.01; Fig. 2).

**Figure 3:**
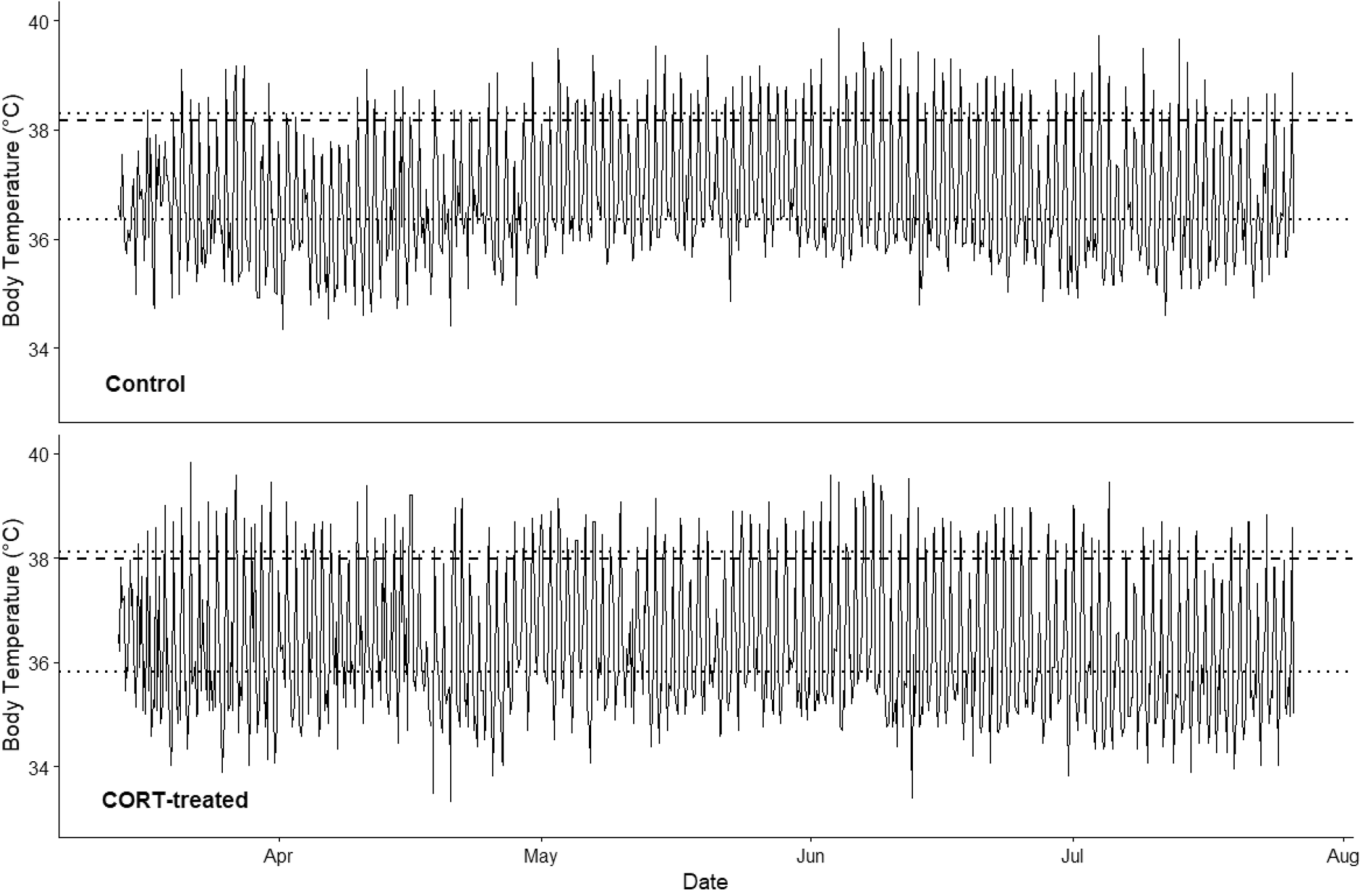
Representative body temperature tracings of banner-tailed kangaroo rats (*Dipodomys spectabilis*) implanted with a silastic implant either impregnated with corticosterone (CORT-treated) or not (Control). Body temperatures (*T*_b_) were recorded every 5 min, but we down sampled to 4-h resolution for this figure. The dashed line represents the modal body temperature (*T*_b-mod_) calculated during the likely active period (defined as 23:00–3:00) for each individual. The range of the dotted lines represent the overall Heterothermy Index (HI) (i.e., not accounting for moonlight or day), positioned according to the relative contribution to HI from readings above and below the *T*_b-mod_. For example, if 95% of the deviation in *T*_b_ occurred below *T*_b-mod_, 95% of the range in HI is below *T*_b-mod_.

**Figure 4:**
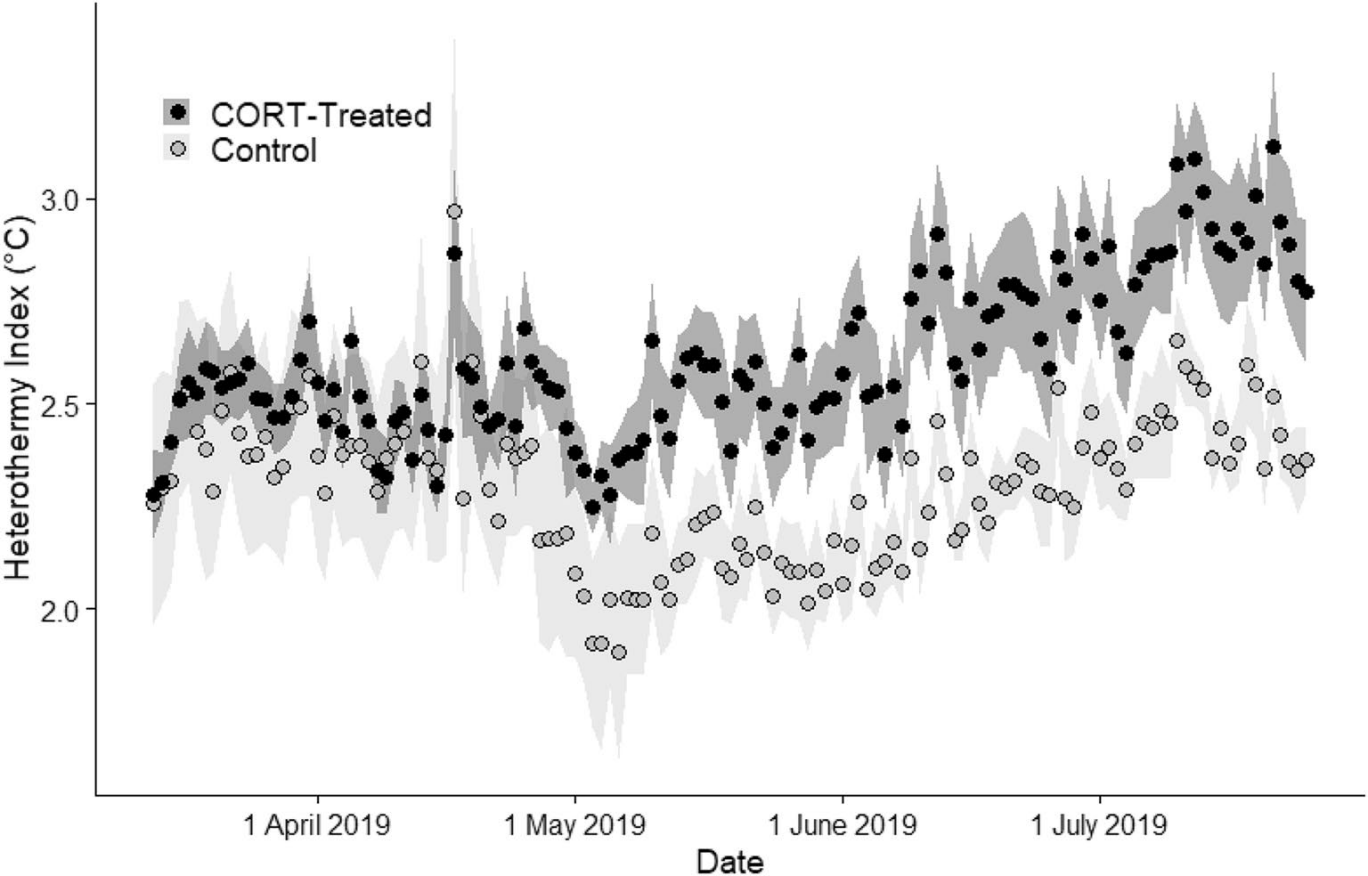
Heterothermy Index (HI) in banner-tailed kangaroo rats (*Dipodomys spectabilis*) implanted with a silastic implant either impregnated with corticosterone (CORT-treated; n = 6) or not (Control; n = 5). The points represent averages of the animals on each day and the shaded areas represent standard error. The large decrease at the beginning of May occurred under a new moon. While we cannot fully explain this decrease, the fact that it occurred in both treatment and control animals suggests the cause is environmental and not experimental. We reanalyzed the data after splitting the dataset into periods before and after 1 May 2019, but the results and interpretation were not changed.

There was no difference in daily *T*_b-mod_ between control (n = 5; 38.1 ± 0.10 °C) and treatment (n = 6; 38.2 ± 0.09 °C) animals (F_1, 9_ = 0.15, *p* = 0.7074; Fig. 3). Heterothermy indices varied among individual animals (F_9, 87.4_ = 16.15, *p* ≤ 0.0001) and increased across the study period (F_1, 98.4_ = 14.5, *p* = 0.0002; Fig. 4). The increasing HI values were largely attributable to increases in time spent below *T*_b-mod_ (which was strongly related to moonlight, see below), but minimum *T*_b_s did also decrease slightly as it became warmer (F_1, 165.7_ = 5.37, *p* = 0.0217). This combination provides strong evidence that *T*_b_ was driven by activity, not environmental temperature. If *T*_b_ was driven by environmental temperatures, we would expect the opposite pattern in both time spent below *T*_b-mod_ and minimum *T*_b_s over the course of the study. After accounting for the general trend of increasing HI values across the study period, CORT-implanted animals had larger HI values than control animals (2.61 ± 0.027 vs. 2.30 ± 0.029; F_1, 87.4_ = 59.16, *p* ≤ 0.0001). Note that HI encapsulates both time and temperature spent away from *T*_b-mod_, so these values should not be interpreted as the absolute difference in *T*_b_ between the two groups. The treatment*day interaction was also significant (F_1, 98.4_ = 12.68, *p* = 0.0006). There was a decrease in HI values in both CORT-treated and control animals around 1 May. We cannot fully explain this decrease, although it did occur right around a new moon. The fact that it happened in both treatment groups suggests the cause was either environmental or perhaps associated with reproduction, and not experimental manipulations. We also reran the models after splitting the dataset before and after 1 May. The general results were similar, and the interpretations did not change, so we discuss only the full model hereafter.

**Figure 5:**
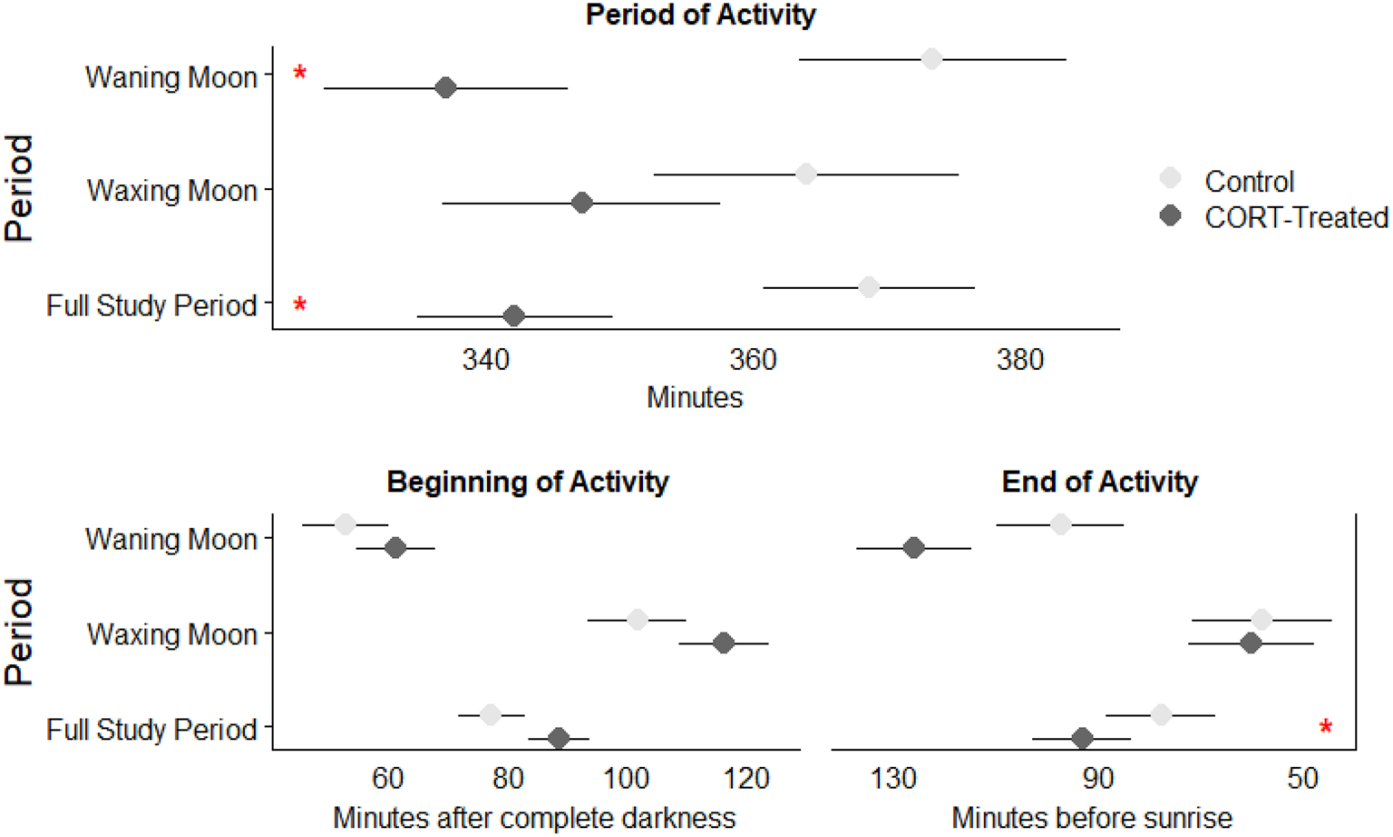
Timing of activity, defined as body temperatures (*T*_b_) within 0.5 °C of modal active body temperature, relative to periods of darkness. For example, control animals raised *T*_b_ near modal active temperatures slightly earlier after both the sun and moon dropped below the horizon (complete darkness) and maintained high *T*_b_ closer to sunrise than CORT-treated animals during the waning phase of the lunar cycle (bottom panels). While neither comparison alone was significantly different, the collective effect was a significantly longer active period for control animals (top panel). Asterisks represent comparisons between control and CORT-treated animals that are significant (*p* < 0.05). Data are presented as least squares means estimates ± standard error.

The activity analysis provides ecological and behavioral context to demonstrate the importance of moonlight in explaining thermoregulatory patterns and why HI values were greater in CORT-implanted animals than control animals. The total time active was nearly identical during the waxing and waning phases of the lunar cycle across all animals (355.5 ± 7.7 min vs. 355.2 ± 6.8 min; F_1, 501_ = 0.00, *p* = 0.09708). However, there was a significant main effect of treatment on activity period, whereby CORT-treated animals reduced daily activity by approximately 26 min compared to control animals (342.1 ± 7.2 min vs. 368.6 ± 7.9 min; F_1, 275_ = 6.14, *p* = 0.0139) across the entire study period (Fig. 5). These differences were largely driven by changes in the end of the activity period when CORT-treated animals cooled below active temperatures about 15 min earlier than control animals (*p* < 0.0001). This pattern was most prominent during waning phases of the lunar cycle, when control animals were active for about 36 min longer than CORT-treated animals (*p* = 0.0076). While not significant, about 30 min of this difference is accounted for at the end of the activity period during the waning phase (when the moon rises at some point during the night), as control animals remained warm longer than CORT-treated animals. Interestingly, while not significant, most of the 16-min difference in activity period during the waxing phase can be accounted for by the beginning of activity (the moon is up and sets during the night). These results are consistent with an increased energy conservation and risk-avoidance behavioral strategy for CORT-treated animals. Somewhat unexpectedly given the apparent shift in foraging behavior, percent fat was not significantly different between CORT treatment (4.0 ± 0.29%) and control (4.6 ± 0.35%) groups at the end of the experiment (F_1, 13_ = 1.87, *p* = 0.1947).

Plasma corticosterone concentrations collected after animals were exposed to the stress of trapping and handling were lower in CORT-treated animals (445.6 ± 166 ng/ml) compared to controls (1871.8 ± 611 ng/ml), hinting at a suppressed stress response in animals chronically exposed to low levels of exogenous CORT (*p* = 0.0672).

## Discussion

Heterothermy in banner-tailed kangaroo rats was more strongly associated with activity periods than environmental temperatures and this pattern was accentuated by disruptions to homeostatic function achieved through CORT implants. CORT-treated animals spent more time with core temperatures below active *T*_b_, even as environmental temperatures increased over the summer. Control animals showed a muted version of the same pattern but were the most homeothermic in the middle of the study. During the latter half of the study period control animals showed increasing heterothermy similar to CORT-treated animals. Unexpectedly, minimum *T*_b_ in banner-tailed kangaroo rats decreased slightly over the summer, as it became warmer. While we would not necessarily predict such a decrease if activity patterns were driving thermoregulatory patterns, we would predict minimum *T*_b_ to *increase* across the study period if environmental temperatures were more important. Thus, the decreasing minimum *T*_b_ further strengthens the conclusion that environmental temperatures played a minor role in thermoregulatory patterns of this nocturnal desert rodent during the warm season. It is becoming increasingly clear that lunar cycles are important in thermoregulation in many nocturnal endotherms. In our study, *T*_b_ tended to be lowest during the day and at night when the moon was above the horizon, presumably as the kangaroo rats remained inside burrows to avoid visual predators. Conversely, in nocturnal visual predators, the pattern is reversed, and *T*_b_ is highest when the moon was bright, indicative of the benefit of moonlight for hunting^56^.

Banner-tailed kangaroo rats, like all desert rodents, are exposed to contrasting environments when in their burrows and above ground. The burrows provide safety from snake, avian, and mammalian predators^16^ and relatively stable temperature and humidity^57^. However, banner-tailed kangaroo rats must expose themselves to predators and occasionally harsh environmental conditions above ground to secure food, guard territories, or find mates^58–60^. Using *T*_b_ as a proxy for activity, as we have done here, does not allow us to directly estimate how much time animals spent above ground. That said, banner-tailed kangaroo rats only maintained active *T*_b_ (defined as *T*_b_ within 0.5 °C of modal *T*_b_) at night, and almost exclusively limited to periods of relative darkness. Given that desert rodents are well known to constrain above-ground activity to periods of relative darkness^21,31,61,62^, we doubt the relationship between *T*_b_ and moon phase is coincidental.

If we take active *T*_b_ to be indicative of the general period when banner-tailed kangaroo rats are active, control animals likely spent more time above ground than CORT-treated animals. Other small desert rodents subjected to experimental CORT treatments increase vigilance or take longer to resume foraging in response to predators encountered during foraging^63,64^. Our results therefore add to the growing body of literature suggesting experimental CORT treatments alter behavior, generally causing animals to adopt risk-adverse strategies. Our results also provide evidence that *T*_b_ might serve as an easily measurable proxy for assessing physiological and behavioral responses to environmental conditions. Advantageously, *T*_b_ can be measured with high temporal resolution for long periods, unlike more traditional measurements of homeostatic responses to potential stressors, like quantification of natural CORT or behavioral metrics.

The effect of the chronic CORT treatment on foraging behavior was especially pronounced under a waning moon (Fig. 5). For comparison, Merriam’s kangaroo rats (*D. merriami*) heavily concentrate aboveground activity in the early, and dark, portion of the night under waning moons^16^. Experimentally increased CORT seems to strengthen this pattern. Banner-tailed kangaroo rats in the treatment group displayed shorter periods of active *T*_b_ compared to control animals that maintained active *T*_b_ later into the night during the waning phase of the lunar cycle, when predation risk increases as the moon rises above the horizon (Fig. 5). While less pronounced, control animals also tended to warm to active *T*_b_ earlier in the night during the waxing phase, when lunar illumination and predation risk are presumably higher than later in the night. Cumulatively, control animals were active longer than CORT-treated animals throughout the study period, however, differences in beginning and ending activity periods alone were not significant within each group between the waning and waxing phases. These results are consistent with other studies that have shown reduced foraging during the waning moon phase^18^. However, they also contradict previous work suggesting the waning phase might be an especially important period for small desert rodents to increase foraging and regain body condition lost during limited foraging under waxing and full moons^65^. If banner-tailed kangaroo rats were losing body condition during the waxing phase like other studied species, and then regaining during the waning phase, we might expect a slow decrease in body condition in CORT-treated animals as they failed to forage enough to regain condition.

CORT treatments did not, however, cause declines in body condition in banner-tailed kangaroo rats. This is surprising for two reasons. First, CORT-treated animals were presumably foraging less along with reduced activity periods, and second, even mildly elevated CORT levels are associated with increased energy demand^66^ and catabolism of lipid stores. However, our results suggest CORT-treated animals were able to behaviorally and/or physiologically compensate for chronic and long-term alteration to HPA functioning caused by the implants. Behavioral compensation could occur via shifts in foraging intensity during active periods or by reliance on food caches to balance out energy lost to decreased foraging above ground. However, experimental CORT treatments caused decreased harvesting rates in Allenby’s gerbils (*Gerbillus andersoni allenbyi*)^63^, and we think increased foraging intensity is an unlikely explanation in our study as well. Thus, we suggest CORT-treated banner-tailed kangaroo rats maintained condition by some combination of reliance on their food cache and maintaining low *T*_b_ for longer periods than control animals.

For small, highly active mammals, even small decreases in core *T*_b_ can have significant energetic benefits because heat loss is determined by the temperature gradient between the core and the environment^67^. Still, increased heterothermy when exposed to low doses of CORT is an interesting response for a larder hoarding species like a kangaroo rat and may offer clues about the relative importance of various fitness-related functions. Eastern chipmunks (*Tamias striatus*), which are similar in size to banner-tailed kangaroo rats and also maintain a larder hoard, decrease use of heterothermy during winter if they have a large food cache^68,69^. While the situation is clearly different in our experiment, one could predict banner-tailed kangaroo rats would take a similar approach and limit heterothermy if their cache is sufficiently large. They might thereby gain the same long-term fitness benefits of territory guarding and mate searching as control animals, but at the expense of consuming seasonally limited hoards. The fact these CORT-treated kangaroo rats relied upon increased heterothermy suggests their hoards potentially contained insufficient food to offset energy lost due to decreases in foraging, or the selective pressure to maintain the hoard for the dry winter season is strong enough to prevent them from consuming their hoard during summer. The underlying physiology is thus key to explaining what could otherwise be interpreted as simple behavioral shifts in response to challenging environmental conditions. The strength of this pattern might also change as climate conditions change over time. During the course of our study, grassland primary production was limited due to drought-like conditions. Drought and more unpredictable precipitation and primary production patterns are becoming more common in the Chihuahuan Desert, impacting rodent populations and their ecological communities^70,71^. If climate change leads to lower, and more unreliable primary productivity, shifts in thermoregulatory behavior might become an increasingly important option for kangaroo rats to balance their energy budget.

Finally, we believe this study demonstrates that experimental manipulation of physiological states has great potential for untangling interactions between the environment, physiological functioning, and behavioral responses^72^.We see two primary benefits to such an approach. First, it avoids some of the common issues with disentangling effects of multiple, often highly correlated, environmental factors on physiologically mediated behaviors by manipulating physiological states directly. Second, and more importantly, it explicitly addresses the possibility that physiology serves as a mechanistic link between environment and behavior instead of the more common view in ecophysiology (taken at least implicitly) that physiology and behavior are best described phenomenologically by measuring them in response to changes in environmental conditions. Clearly, there are limitations to pharmacological manipulations of physiological function, but they offer a convenient way to gain mechanistic understanding of behaviors under some conditions.

The diverse and interrelated processes necessary to maintain homeostasis of energy, water, and body temperature are vital in mediating organismal responses to environmental conditions. Given the rapidly changing environment faced by animals, it will become increasingly important to develop a mechanistic understanding of how functioning of these physiological processes is manifested in behavior, survival, and reproduction. Here, we demonstrated a change in thermoregulatory precision likely caused by a change in behavior (i.e., time spent active) by banner-tailed kangaroo rats in response to temporally variable foraging conditions (i.e., darkness). We further demonstrated that a mild, but chronic physiological hormonal signal often associated with stress strengthened the effect of background ecological patterns, making them more easily detectable. We encourage other researchers to use similar manipulations to determine how other physiological functions affect behavior and how an animal interacts with its environment.

## Supplementary Information

Supplementary Information.

## Data Availability

The final dataset is available on Data Dryad https://doi.org/10.5061/dryad.ghx3ffbn1.
