# Supplementary material for: Heterothermy as a mechanism to offset energetic costs of environmental and homeostatic perturbations
Source: Sci Rep. 2021 Sep 24;11:19038. doi: 10.1038/s41598-021-96828-0 (PMC8463709; doi:10.1038/s41598-021-96828-0)
Supplement: Supplementary file 1 — Supplementary Information. [file 41598_2021_96828_MOESM1_ESM.docx]

**In vitro test of silastic implants**

We quantified CORT release rates from silastic implants using an *in vitro* assay ^1,2^. We placed silastic rods into 10 ml of PBS buffer, stored at 37˚C for 42 days, and sampled on days 1, 7, 14, 28, and 42. We replaced the storage buffer with fresh PBS every 3 days. On sampling days, we placed the implants into 1 ml of fresh PBS in eppendorf tubes for 6 hours at 37˚C, after which the rods were returned to their long-term storage tubes with 10 ml of fresh PBS. The 1 ml samples remained frozen at -20˚C until we conducted analysis by ELISA (DetectX^®^ Corticosterone EIA Kit, Arbor Assay, Ann Arbor, MI, USA). Serially diluted samples demonstrated extremely high concentrations of CORT were released into solution requiring that we dilute the samples by 10^4^ for the assay. We ran all samples on one plate. The implants initially released a high dose of CORT on day 1 (28 ± 0.5 µg/cm/6 hours; Fig. S1), but decayed to a more stable release rate of approximately 9.3 ± 0.9 µg/cm/6 hours after week 2. This pattern of a pulse of high release followed by decline to a more stable release rate is similar to previously published reports ^1,2^. We suspect that rapid removal of a compound from the surface or near the surface of a cylinder likely produces the initially high concentration release. We estimated the sustained daily release of CORT reported in the paper (36 µg/cm/day) from the 6-hour results for days 14 to 42, in order to compare to previous reports.


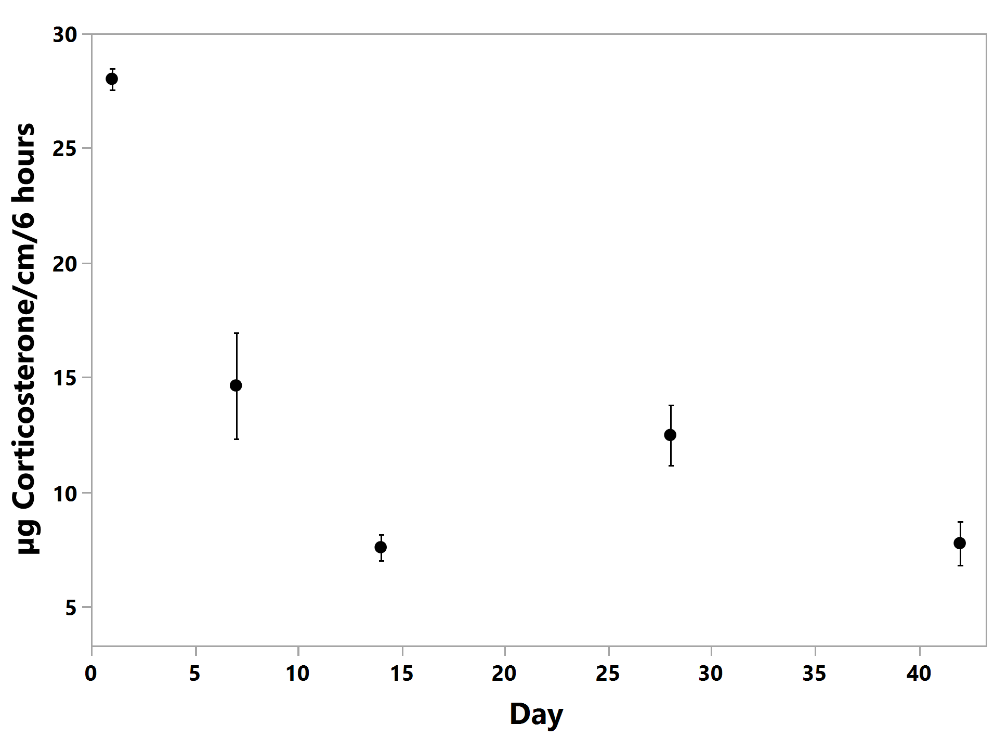


Figure S1. *In vitro* release of corticosterone from silastic implants across 42 days. We stored samples in 10ml of PBS buffer at 37˚C, and changed the PBS every 3 days. On sample days, we placed the implants into 1 ml of fresh buffer for 6 hours at 37˚C to obtain samples.

References

1. Gupta, R. C. *et al.* Controlled-release systemic delivery - a new concept in cancer chemoprevention. *Carcinogenesis* **33**, 1608–1615 (2012).

2. Park, J. *et al.* Local delivery of hormonal therapy with silastic tubing for prevention and treatment of breast cancer. *Sci. Rep.* **8**, 1–12 (2018).
